# A Rare Case of Hypertrophic Cardiomyopathy with Subendocardial Late Gadolinium Enhancement in an Apical Aneurysm with Thrombus

**DOI:** 10.1155/2014/780840

**Published:** 2014-06-25

**Authors:** Yusuke Morita, Takao Kato, Mitsumasa Okano, Kanae Su, Masahiro Kimura, Eri Minamino, Eisaku Nakane, Toshiaki Izumi, Shoichi Miyamoto, Tetsuya Haruna, Moriaki Inoko

**Affiliations:** Cardiovascular Center, Tazuke Kofukai Medical Research Institute, Kitano Hospital, 2-2-20 Ogimachi, Kita-ku, Osaka 530-8480, Japan

## Abstract

The mechanisms responsible for the development of apical aneurysms in cases of hypertrophic cardiomyopathy (HCM) are currently unclear but likely involve multiple factors. Here, we present a case of HCM with marked subendocardial fibrosis involving the apical and proximal portions of the left ventricle. A 71-year-old man with left ventricular hypertrophy presented with signs and symptoms of heart failure. The presence of asymmetrical left ventricular hypertrophy and bilateral, thickened ventricular walls with an apical aneurysm on transthoracic echocardiography suggested a diagnosis of HCM with ventricular dysfunction. No intraventricular pressure gradients with obstruction were identified. Late gadolinium enhancement (LGE) with cardiac magnetic resonance imaging and endomyocardial biopsies showed subendocardial fibrosis involving the apical aneurysm and proximal portion. Whereas LGE in a transmural pattern is commonly observed in HCM apical aneurysms, subendocardial LGE, as noted in the present case, is a relatively rare occurrence. Thus, the present case may provide unique insights into the adverse remodeling process and formation of apical aneurysms in cases of HCM.

## 1. Introduction

The life-long process of left ventricular remodeling and progressive dysfunction occur in a substantial proportion of patients with hypertrophic cardiomyopathy (HCM), of which a subset presents with extreme fibrosis and may progress to heart failure and sudden cardiac death [1]. Due to the slow progression and heterogeneous nature of HCM, the exact process of adverse left ventricular remodeling is currently not fully understood and probably reflects the interplay of microvascular ischemia, cardiomyocyte energy depletion, and apoptosis, leading to progressive myocyte loss and fibrotic replacement of the myocardium [2].

Adverse left ventricular remodeling in HCM is characterized by variable patterns of myocardial fibrosis, visualized by cardiac magnetic resonance (CMR) imaging as late gadolinium enhancement (LGE). There is vast support for the notion that LGE likely constitutes areas of myocardial replacement fibrosis [3]. In cases of HCM, LGE generally shows a typical midwall localization with sparing of the subendocardial region, but transmural patterns may be observed in severe cases [4]. Here, we present a rare case of global subendocardial fibrosis in an apical aneurysm and the proximal portion of the left ventricle (LV), identified by CMR imaging and endomyocardial biopsy. This case may provide unique insights into the adverse remodeling process and formation of apical aneurysms in cases of HCM.

## 2. Case Presentation

**Figure 1:**
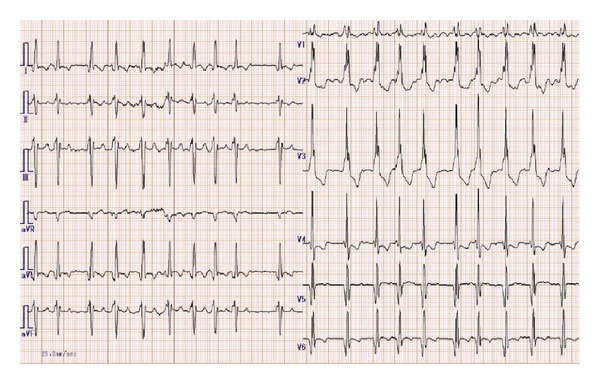
The 12 lead electrocardiograms on admission showing atrial fibrillation with rapid ventricular response and complete right bundle branch block.

**Figure 2:**
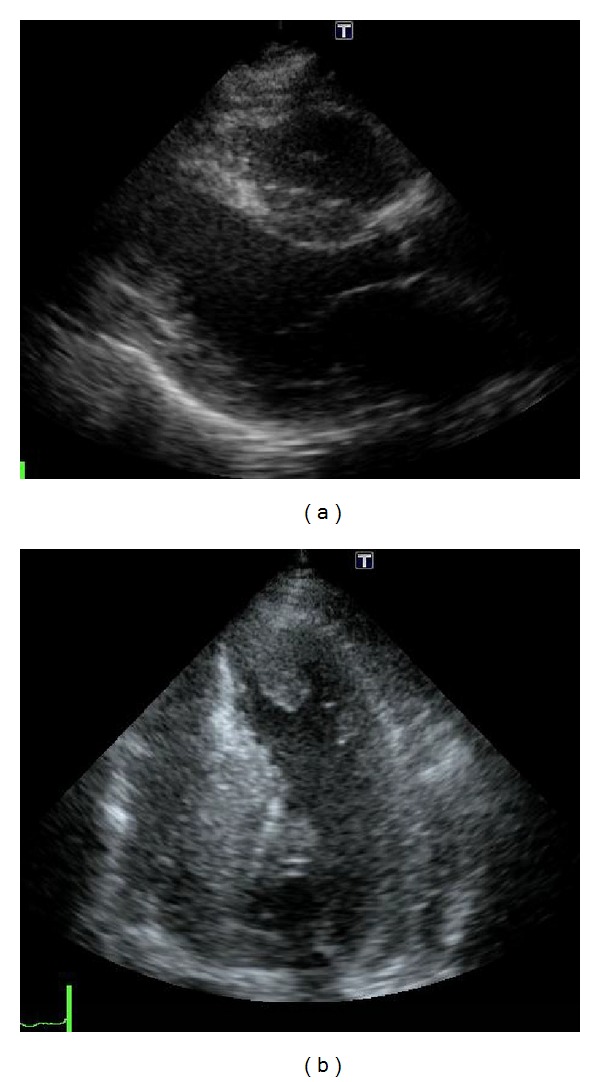
Parasternal long-axial view (a) and four-chamber view (b) of the transthoracic echocardiography showing asymmetrical hypertrophy and a thin-walled apical aneurysm with thrombus.

A 71-year-old man developed progressive dyspnea, orthopnea, general malaise, and edema in both the lower limbs. He was admitted to a local hospital for congestive heart failure, categorized as New York Heart Association functional class III, and atrial fibrillation with a rapid ventricular response. He had no history of hypertension, diabetes, or familial cardiac disease. An electrocardiogram showed atrial fibrillation with a rapid ventricular response and a complete right bundle branch block (Figure 1). Low voltage, a typical finding in cardiac amyloidosis, was not observed. Laboratory findings revealed highly elevated levels of B-type natriuretic peptide (1272.6 pg/mL; normal range, <18.4 pg/mL) and troponin I (0.491 ng/mL; normal range, <0.045 ng/mL). The white blood cell (WBC) count was 5,830/*μ*L, with 1.5% eosinophils, and the C-reactive protein level was 0.48 mg/dL. Transthoracic echocardiography revealed an extensive, thin-walled akinetic region and thrombus at the apex, hypokinesis of the basal and midportion of the LV, and a decreased LV ejection fraction of 37% (Figure 2). The LV diastolic and systolic diameters, LV posterior and septal wall thicknesses, and right ventricle (RV) free wall thickness were 49 mm, 39 mm, 12 mm, 17 mm, and 10 mm, respectively. There was no evidence of left ventricular outflow tract obstruction, systolic anterior motion of the mitral valve, or aortic valve disease.

**Figure 3:**
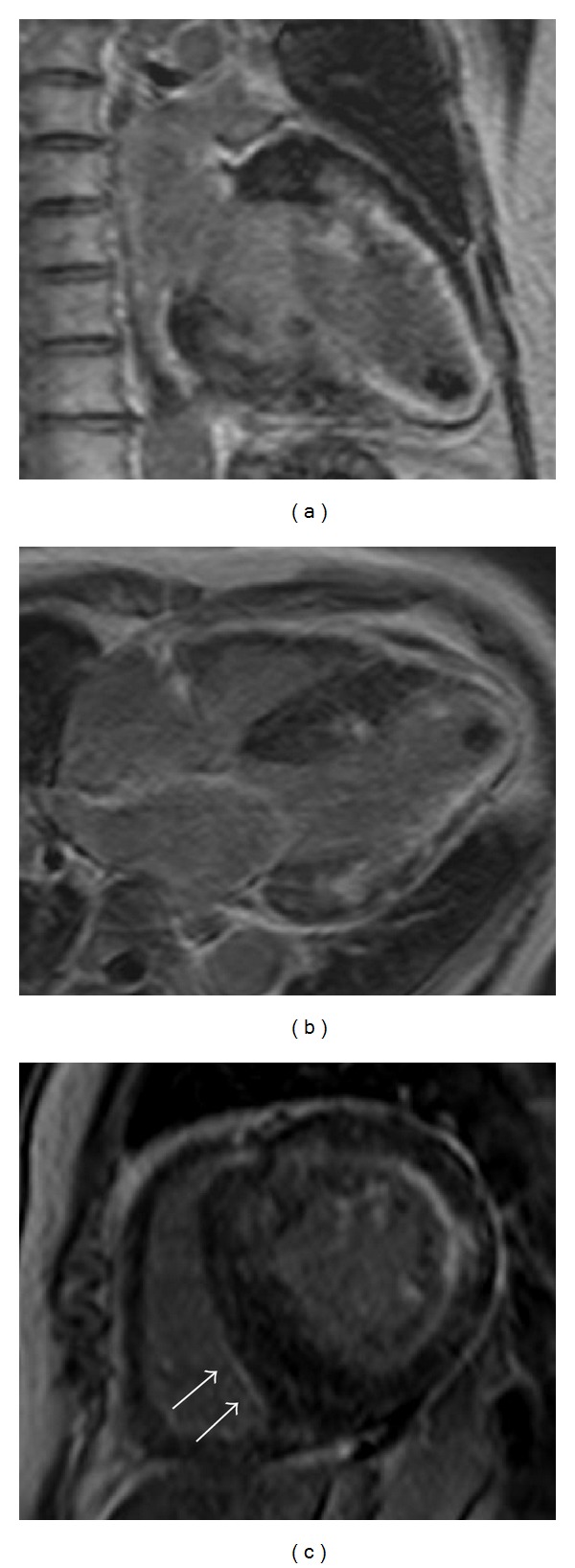
The long-axis view (a), four-chamber view (b), and short axis view (c) of cardiac magnetic resonance imaging revealed a thin-walled apical aneurysm, with subendocardial late gadolinium enhancement (LGE) in an extensive area of the mid-to-apex region of the left ventricle and in a smaller area of the right ventricle (small arrows). LGE involved the majority of the basal lateral wall. Smaller midmyocardial LGE located in the basal interventricular septum, the wall of which was markedly thickened, was also observed. Scanner type: 3D-FFE, matrix scan: 224, flip angle: 15 degrees, and bandwidth: 324.7 Hz. The repetition time, echo time, and inversion time were (a) 3.910, 1.219, and 280 m/s, (b) 4.005, 1.250, and 290 m/s, and (c) 4.078, 1.272, and 300 m/s, respectively.

The long-axis (Figure 3(a)), four-chamber (Figure 3(b)), and short axis views (Figure 3(c)) of the CMR images revealed a thin-walled, apical aneurysm, with subendocardial LGE in an extensive area of the mid-to-apex region of the LV and a smaller area in the RV. The LGE involved the majority of the basal lateral wall. Smaller, midmyocardial LGE was observed in the basal interventricular septum, of which the wall was markedly thickened.

**Figure 4:**
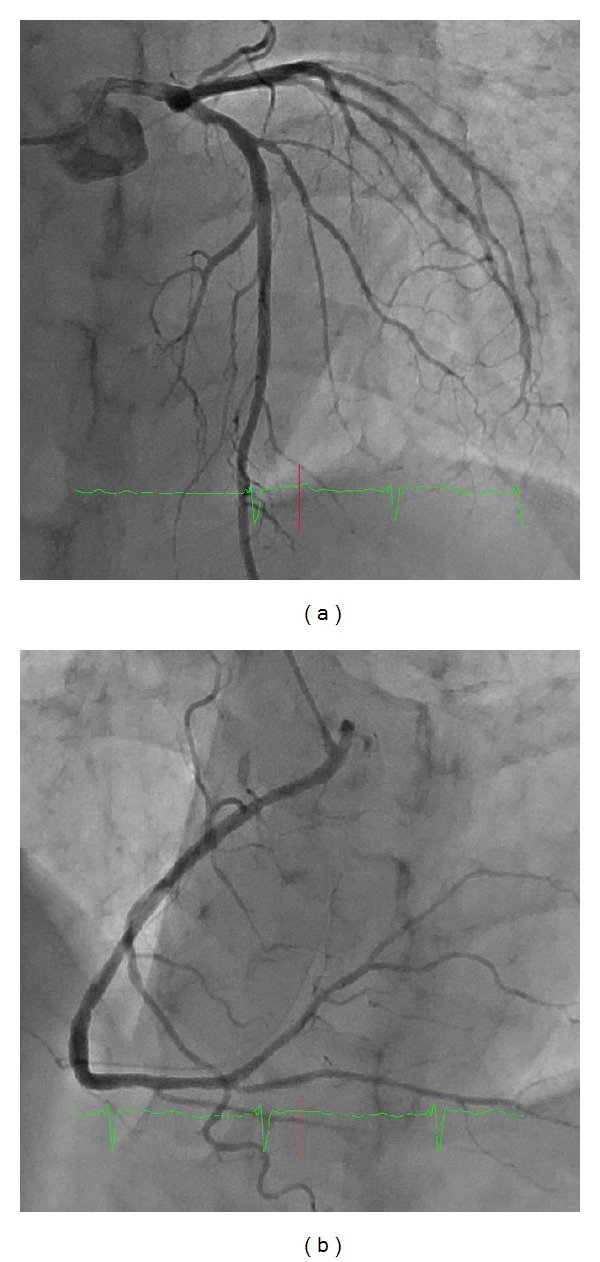
Coronary angiography showed no significant stenosis in the left (a) and right (b) epicardial arteries. Myocardial bridging was not observed.

**Figure 5:**
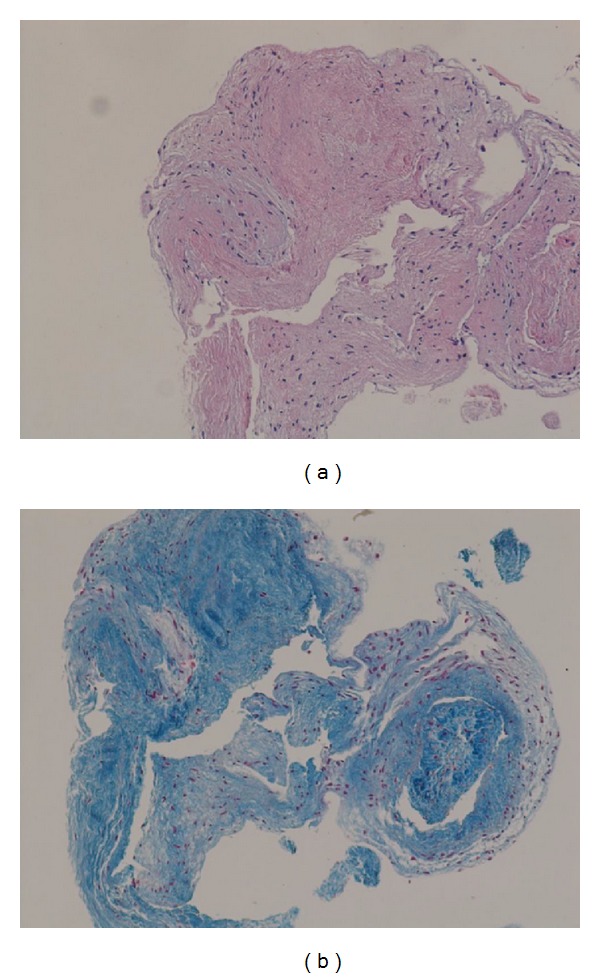
Histological findings. Hematoxylin and eosin staining, 10x magnification (a), and Azan staining, 10x magnification (b), of endomyocardial biopsies from the right ventricular septum. Thickened fibrous endomyocardium extended through all layers of each specimen.

Regional subendocardial fibrosis is frequently observed in patients with subendocardial infarction. However, in the present case, coronary angiography did not show coronary stenosis or myocardial bridging (Figure 4). Three endomyocardial biopsy specimens were collected from the right ventricular septum and indicated that, in each sample, the thickened fibrous endomyocardium extended through all layers (Figure 5). We considered performing a left ventricular examination, but an apical thrombus in the LV was recognized as the source of embolic material and left ventricular biopsy and pressure measurement were not performed. The patient was treated with diuretics, bisoprolol, losartan, warfarin, and spironolactone, resulting in improvements in the heart failure symptoms.

## 3. Discussion

### 3.1. Diagnosis of HCM

A clinical diagnosis of HCM is conventionally made based on a LV wall thickness >15 mm upon CMR imaging in the absence of other plausible underlying causes [5]. Accordingly, in the present case, the markedly thickened bilateral ventricular walls and asymmetrical hypertrophy of the LV in the absence of another disease capable of producing hypertrophy, such as hypertension or aortic stenosis, were consistent with a diagnosis of HCM. In addition, genetic testing is an effective tool for the definitive diagnosis of HCM and identification of affected family members [5]; however, this was not performed in the present case.

Apical aneurysms are well recognized as a complication of myocardial infarction and usually involve the anterior wall supplied by the left descending coronary artery. Due to the normal coronary arteries in our patient, vasospastic angina and coronary thrombosis with atrial fibrillation were initially suspected as a possible explanation. However, diffuse hypokinesis of the proximal portion and LGE involving a large territory of the mid-to-apex region of the LV ruled out this diagnosis.

### 3.2. Mechanism behind the Formation of the Apical Aneurysm in HCM

HCM patients with apical aneurysms represent a unique subgroup, accounting for only 2% of all reported cases by 2-dimensional echocardiography. However, given that CMR is superior to 2-dimensional echocardiography regarding the identification of the presence of apical aneurysms and the assessment of LV hypertrophy, apical aneurysms are potentially underdiagnosed [6]. These patients are considered high-risk patients due to their increased risks of adverse clinical events, including evolution into end-stage phase, arrhythmic sudden death, and stroke. Approximately 36% of apical aneurysm cases are found in conjunction with midventricular obstruction and intraventricular pressure gradients [1]. Moreover, several studies have found that apical hypertrophy, which is predominantly characterized by myocardial hypertrophy in the apex, is associated with the occurrence of apical aneurysms with midventricular obstruction [7, 8]. In brief, the elevated pressure may exceed the diastolic coronary blood flow due to the obstruction, resulting in circumferential apical scarring and thinning over time. However, since apical aneurysms can also be identified in cases of HCM without obstruction or intraventricular pressure gradients, as in our case, the mechanisms responsible for their formation are currently unclear.

Cases of HCM with obstruction and preserved contractility have been previously reported to progress into apical aneurysms and are occasionally accompanied by diminished intraventricular gradients due to ventricular wall motion abnormalities [9]. The present case showed global left ventricular wall motion abnormalities and an apical aneurysm without intraventricular gradients.

The pattern of fibrosis in our case involved the apical and proximal portions of the subendocardium. The extent of the fibrosis, as demonstrated by LGE localization, was similar among the apical and proximal portions, implying that the remaining viable myocardium was thinner in the apical portion than in the proximal portion. We speculate that this may represent one of the mechanisms behind the formation of apical aneurysms without obstruction or intraventricular pressure gradients, although the previous findings at an earlier stage of the disease were unidentified in our case.

### 3.3. Subendocardial LGE and Differential Diagnosis

LGE in HCM is most frequently located in the ventricular septum and LV free wall, appearing as small, punctuate, and patchy midwall hyperenhancement [4, 10]. LGE of apical aneurysms in HCM has been commonly described as a transmural pattern [4]; however, subendocardial LGE, as observed in the present case, is a relatively rare occurrence [9]. The vasodilator response and coronary flow reserve are impaired in HCM, particularly in the subendocardial layers due to high LV end-diastolic pressure [11]. Subendocardial predisposition to ischemia and fibrotic replacement may explain the subendocardial LGE in the present case.

Continuous subendocardial LGE is also observed in endomyocardial fibrosis, which is a disease entity characterized by a massive, fibrotic tissue deposition in the subendocardial layer of one or both ventricles [12, 13] and is the most common restrictive cardiomyopathy worldwide [13]. A previous case report describing the rare association of subendocardial LGE in endomyocardial fibrosis and typical histopathological findings of HCM described myocardial hypertrophy of the interventricular septum and disarray [14]. Subendocardial fibrosis predominantly involves the ventricular apex in endomyocardial fibrosis, but the underlying causes and mechanisms are currently not well understood. However, it has been reported that poor vascularization at the apex may result in failure of cardiac cell repair, subendocardial degeneration, and fibrosis [12]. The most important features for the diagnosis of endomyocardial fibrosis are a reduction in longitudinal diameter and obliteration of the apex with normal-size LV. In addition, despite the fact that the roles of cardiovascular allergy and infectious agents in the pathogenesis of endomyocardial fibrosis are controversial, hypereosinophilia is found in up to 30% of patients [12, 13], although this was not observed in the present case.

Lastly, similarly to HCM and endomyocardial fibrosis, advanced amyloidosis and sarcoidosis can also cause subendocardial LGE [15] and should be considered as differential diagnoses, depending on the clinical context. Multiorgan disorders, such as nephrotic syndrome and autonomic neuropathy, low voltage on an electrocardiogram (defined as all limb leads <5 mm in height), and speckled sparkling signs on echocardiography are frequently observed in amyloid light-chain amyloidosis, but there were no such findings in our case [16]. Furthermore, right bundle branch block (RBBB) is found in 3.6% of patients with amyloid light-chain amyloidosis [17] and in 9% of patients with hypertrophic cardiomyopathy [18], and negative T waves with RBBB in the left precordial leads (aVL and V4–6 leads) are also compatible with the diagnosis of HCM in the present case [19].

## 4. Conclusion

CMR provides detailed information regarding ventricular morphology and function in HCM patients. Our HCM case demonstrated incidental findings of subendocardial LGE, which mainly allowed the evaluation of myocardial fibrosis, observed in an apical aneurysm and the proximal portion of the LV. Although more data are needed on this topic, our findings suggest that CMR may be a useful, noninvasive tool for the evaluation of the underlying pathophysiological mechanisms in adverse left ventricular remodeling in HCM patients.

## References

[1] Maron MS, Finley JJ, Bos JM (2008). Prevalence, clinical significance, and natural history of left ventricular apical aneurysms in hypertrophic cardiomyopathy. *Circulation*.

[2] Watkins H, Ashrafian H, Redwood C (2011). Inherited cardiomyopathies. *The New England Journal of Medicine*.

[3] Sotgia B, Sciagrà R, Olivotto I (2008). Spatial relationship between coronary microvascular dysfunction and delayed contrast enhancement in patients with hypertrophic cardiomyopathy. *Journal of Nuclear Medicine*.

[4] Maron MS, Appelbaum E, Harrigan CJ (2008). Clinical profile and significance of delayed enhancement in hypertrophic cardiomyopathy. *Circulation Heart Failure*.

[5] Gersh BJ, Maron BJ, Bonow RO (2011). 2011 ACCF/AHA guideline for the diagnosis and treatment of hypertrophic cardiomyopathy: executive summary: a report of the American College of cardiology foundation/American heart association task force on practice guidelines. *Circulation*.

[6] Maron BJ, Maron MS (2013). Hypertrophic cardiomyopathy. *The Lancet*.

[7] Matsubara K, Nakamura T, Kuribayashi T, Azuma A, Nakagawa M (2003). Sustained cavity obliteration and apical aneurysm formation in apical hypertrophic cardiomyopathy. *Journal of the American College of Cardiology*.

[8] Lazaros G, Kouvousis N, Kotsanis A, Matsakas E (2007). Apical hypertrophic cardiomyopathy with midventricular obstruction and apical aneurysm. *International Journal of Cardiology*.

[9] Fighali S, Krajcer Z, Edelman S, Leachman RD (1987). Progression of hypertrophic cardiomyopathy into a hypokinetic left ventricle: higher incidence in patients with midventricular obstruction. *Journal of the American College of Cardiology*.

[10] Noureldin RA, Liu S, Nacif MS (2012). The diagnosis of hypertrophic cardiomyopathy by cardiovascular magnetic resonance. *Journal of Cardiovascular Magnetic Resonance*.

[11] Petersen SE, Jerosch-Herold M, Hudsmith LE (2007). Evidence for microvascular dysfunction in hypertrophic cardiomyopathy: new insights from multiparametric magnetic resonance imaging. *Circulation*.

[12] Mocumbi AO, Ferreira MB, Sidi D, Yacoub MH (2008). A population study of endomyocardial fibrosis in a rural area of Mozambique. *The New England Journal of Medicine*.

[13] Mocumbi AOH, Falase AO (2013). Recent advances in the epidemiology, diagnosis and treatment of endomyocardial fibrosis in Africa. *Heart*.

[14] Salemi VMC, D'Andretta Iglezias S, Benvenuti LA (2012). An unusual association of endomyocardial fibrosis and hypertrophic cardiomyopathy in a patient with heart failure. *Cardiovascular Pathology*.

[15] Maceira AM, Joshi J, Prasad SK (2005). Cardiovascular magnetic resonance in cardiac amyloidosis. *Circulation*.

[16] Murtagh B, Hammill SC, Gertz MA, Kyle RA, Tajik AJ, Grogan M (2005). Electrocardiographic findings in primary systemic amyloidosis and biopsy-proven cardiac involvement. *The American Journal of Cardiology*.

[17] Dubrey SW, Cha K, Anderson J (1998). The clinical features of immunoglobulin light-chain (AL) amyloidosis with heart involvement. *Oxford Journals Medicine*.

[18] Delcrè SDL, Di Donna P, Leuzzi S (2013). Relationship of ECG findings to phenotypic expression in patients with hypertrophic cardiomyopathy: a cardiac magnetic resonance study. *International Journal of Cardiology*.

[19] Suzuki J, Shin WS, Shimamoto R (1999). Clinical implication of left precordial T wave inversions in the presence of complete right bundle branch block. *Japanese Heart Journal*.

